# Hydrogels as a new platform of therapeutic systems in oncological treatment - use as a protective barrier and drug carriers

**DOI:** 10.3389/fbioe.2025.1673253

**Published:** 2025-09-23

**Authors:** Katarzyna Czerwiec, Weronika Szczecińska, Michał Pikuła

**Affiliations:** ^1^ Division of Clinical Anatomy, Laboratory of Tissue Engineering and Regenerative Medicine, Medical University of Gdansk, Gdansk, Poland; ^2^ The Department of General Surgery, Hospital Copernicus in Gdansk, Gdansk, Poland; ^3^ Laboratory of Tissue Engineering and Regenerative Medicine, Department of Embryology, Medical University of Gdansk, Gdansk, Poland; ^4^ Department of Biochemistry, University of Physical Education and Sport, Gdańsk, Poland

**Keywords:** hydrogels, oncology, alginate, chitosan, hyaluronic acid, peptide hydrogels

## Abstract

Hydrogels as three-dimensional polymer networks capable of reversibly absorbing water are of increasing interest among researchers. Hydrogels, especially those of natural origin such as alginate, chitosan, hyaluronic acid, peptide hydrogels, thanks to properties such as biocompatibility, biodegradability, bioactivity, can serve as an effective protective barrier or drug carrier. Thanks to the possibility of their modification, they can be an innovative platform supporting anticancer treatment. The examples presented in this publication confirm that these products can increase the effectiveness of treatment and reduce the effects of side effects.

## 1 Introduction

One of the greatest socio-economic problems of the 21st century is cancer. It causes 1 in 6 deaths worldwide. This disease constitutes a significant obstacle to extending life expectancy, but also a serious problem related to social and macroeconomic costs, which vary depending on the type of cancer, geographical location or gender (Bray et al., 2024). The global economic cost of cancer between 2020 and 2050 is estimated to be $25.2 trillion (in international dollars). Cancers with the highest economic costs include: cancer of the trachea, bronchi and lungs, cancer of the colon and rectum, breast cancer, liver cancer, and leukaemia (Chen et al., 2023). Cancer is a disease that originates from normal body tissues. But, due to persistent pathological features, it grows in an uncontrolled manner and is not susceptible to factors regulating cell growth, maturation and function (Brown et al., 2023). In the development of this disease, there is a change in cellular metabolism, which meets the energy and biosynthetic needs of uncontrolled proliferation of cancer cells (Xu et al., 2023).

Radiotherapy, chemotherapy and surgery play an essential role in the treatment of cancer individually or in combination. Cancer treatment is evolving, which is associated with technical progress in surgery, radiotherapy, as well as the discovery of new drugs such as cell cycle checkpoint inhibitors, immune response modifiers: CAR-T immunotherapy, anti-PD-1 and anti-PD-L1 antibody therapy (Mee et al., 2023). The type of cancer, its location and the stage of cancer advancement determined by the TNM classification allow us to choose the best treatment option and its progression (Rosen and Sapra, 2025). One of the methods of early prevention of this disease is prophylaxis. We are unable to determine one specific factor responsible for the development of cancer, because usually a number of factors related to addictions, lifestyle and the surrounding environment are involved in the development of cancer (Vineis and Wild, 2014; Collatuzzo and Boffetta, 2023). It is extremely important to perform preventive tests that will allow for the observation of disturbing changes at their early, non-advanced stage, as well as annual check-ups in people at increased risk of hereditary cancers.

**FIGURE 1:**
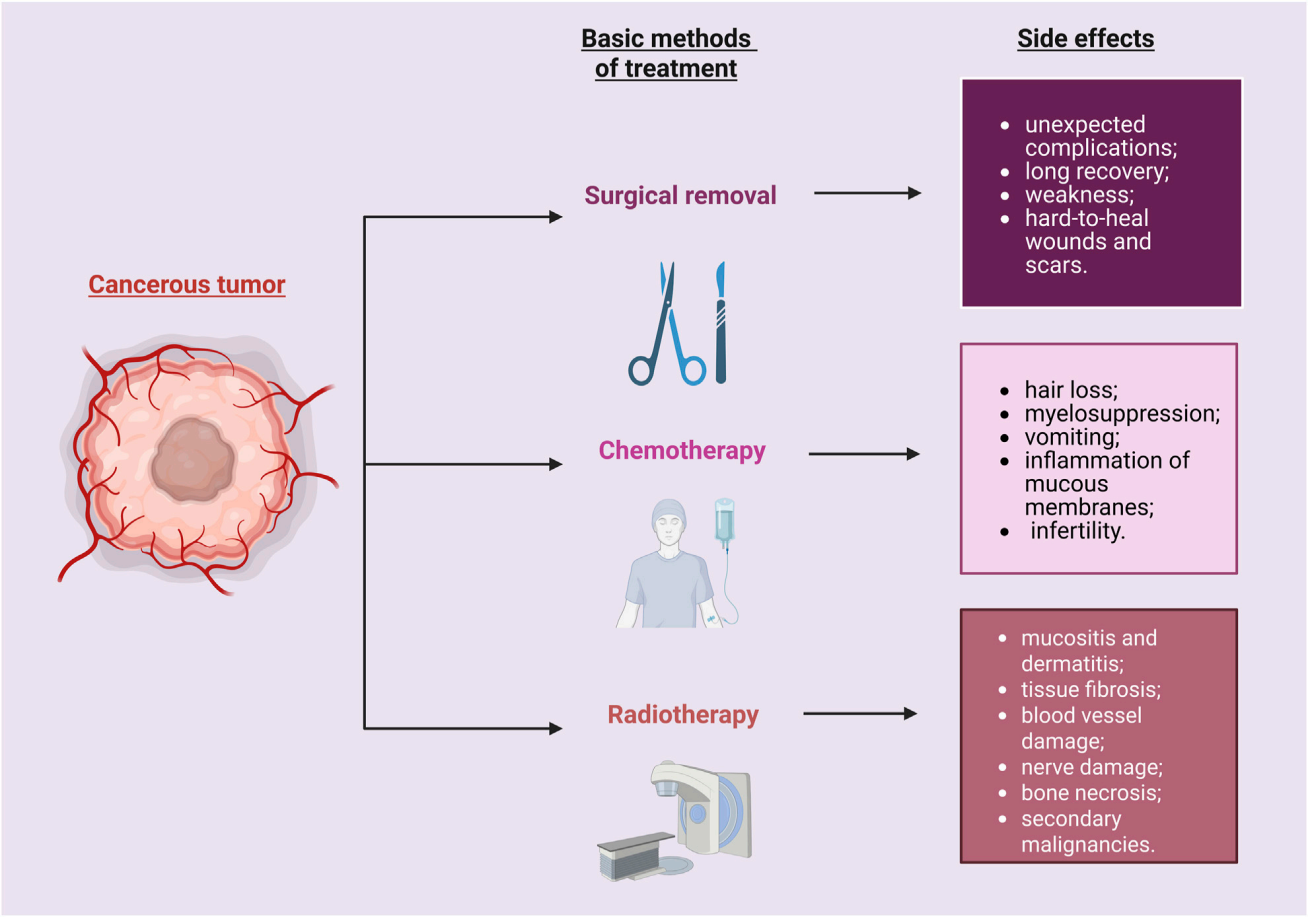
Cancer therapy may cause various side effects. They may impact the patient’s recovery process. Created with BioRender.com.

Since any treatment can cause side effects, the treatment of cancer is no different (Figure 1). It is important to remember that each person may respond differently to the treatment given. The purpose of chemotherapy is to inhibit cell proliferation and multiplication to avoid the spread of abnormal cells and prevent metastasis to other organs. Chemotherapy preparations interfere with the synthesis of RNA, DNA or proteins (Sun Y. et al., 2021). When the chemotherapy drug works properly, cell death occurs or programmed cell death is triggered by apoptosis. The effects of chemotherapy are a reflection of their mechanisms of action. Drugs administered during chemotherapy affect rapidly multiplying cells, hair follicles, bone marrow and the digestive tract, therefore undesirable effects may include: excessive hair loss, myelosuppression, vomiting, inflammation of mucous membranes, other adverse effects on the digestive system, and infertility (Amjad et al., 2025; Katta et al., 2023). It should be emphasized that in the case of chemotherapy, due to its non-selectivity, the toxic effect affects not only abnormal cells, but also those that are healthy. The bioavailability of these chemotherapy drugs is poor for cancer tissues, so higher doses of chemotherapeutics are needed, resulting in increased toxicity in healthy cells. Multidrug resistance may also occur in such conditions (Senapati et al., 2018). Side effects affecting healthy tissue are the cause of high mortality among cancer patients. New solutions are being sought that will enable the delivery of an optimal dose of chemotherapy drug so that it has the least possible impact on healthy cells.

Radiotherapy is a treatment method that uses high-energy rays or radioactive substances to damage cancer cells and stop them from growing and dividing (Gianfaldoni et al., 2017). High frequency waves cause ionization in the tissue. Ionization occurs as a result of an electron being knocked out of the atomic orbit by an electromagnetic wave. Free electrons cause the formation of free radicals – unstable molecules with high chemical reactivity (Tumilaar et al., 2024). The DNA of the cell nucleus is a structure susceptible to damage as a result of ionization. DNA is damaged when a free electron hits the DNA strand and as a result of the action of free radicals. Ionizing radiation damages cancer cells more effectively than normal cells. Radiotherapy is by nature a conservative treatment – radiation treatments are painless and bloodless, but they last relatively long. The side effects of radiotherapy depend primarily on the location of the tumour and the dose of radiation received (Barazzuol et al., 2020). During radiotherapy, the skin is particularly exposed to the effects. The earliest adverse events of radiotherapy include mucositis and dermatitis (Iacovelli et al., 2020). Over time, tissue fibrosis, blood vessel damage, nerve damage, bone necrosis, and secondary malignancies may occur (Li et al., 2023). From a trivial inconvenience it can develop into a life-threatening situation. Radiation-induced mucositis first begins with acute inflammation of the mucosa, tongue, and throat after exposure to radiation (Rao et al., 2021). During this time, a cascade of immune reactions occurs, including recruitment of immune cells, release of inflammatory cytokines, chemotactic mediators, and growth factors. The described condition can progress to an acute stage, where food and water intake is prevented, which is associated with weight loss, and also a septic complication, which is a consequence of protective epithelial barriers and the basement membrane (Maria et al., 2017).

**FIGURE 2:**
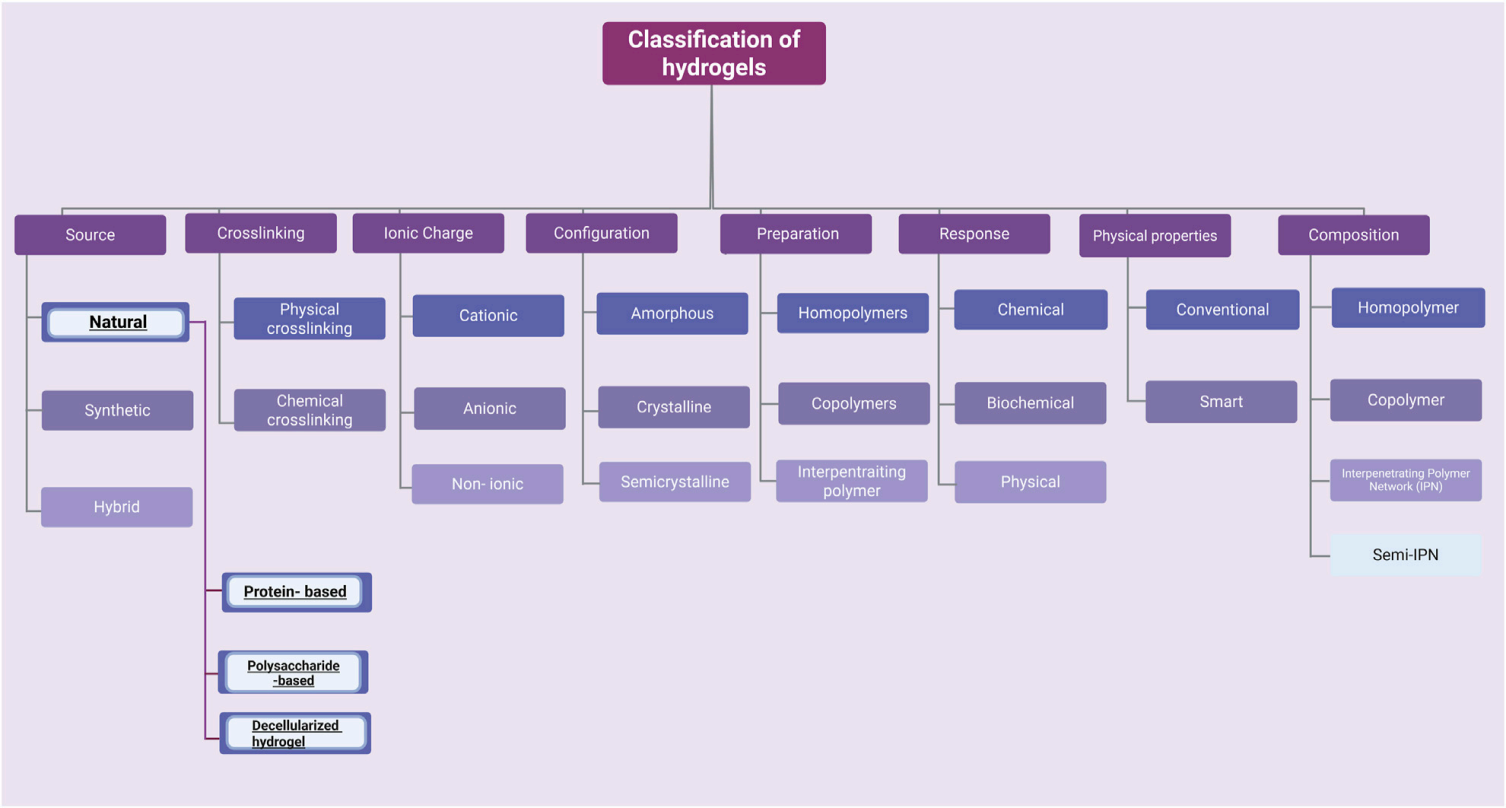
There are many possibilities for classifying hydrogels. This article focuses only on a few examples of naturally derived hydrogels. Created with BioRender.com.

Currently, advanced techniques and products are being sought that reduce the risk of negative effects caused by radiotherapy and chemotherapy. Hydrogel is a three-dimensional network of hydrophilic polymer matrices that are able to retain a large amount of water (>10%) (Ho et al., 2022; Zhao et al., 2023; Lee et al., 2023). Crosslinking, which arises between hydrophilic matrices, maintains a constant, stable structure that is not dissolved in the aquatic environment. The unique property of this group of materials - the ability to absorb large amounts of water is caused by the presence of functional groups in their structure such as: OH, -COOH, -CONH, -NH_2_, -SO_3_H (Dodda et al., 2023). There are many classifications of hydrogels due to their different properties: chemical, physical or source of origin (Bashir et al., 2020; Bustamante-Torres et al., 2021; Khan et al., 2024) (Figure 2). One way to classify hydrogels is by cross-linking. Cross-linking is the process by which covalent or ionic bonds are formed between polymer chains. This process creates a network structure responsible for the mechanical properties of the hydrogel. This allows for modifications of polymers and biomolecules, which promote specific hydrogel characteristics. There are two methods of hydrogel cross-linking: chemical and physical (Akhtar et al., 2016). The formation of covalent bonds between polymer chains is commonly described as chemical cross-linking. The reaction requires a cross-linking agent that initiates and forms the bonds (Alavarse et al., 2022). The covalent bond between polymers is characterized by durability (compared to physical cross-linking), and the reaction product itself is characterized by increased stability in a physiological environment and desirable mechanical properties. Several chemical cross-linking methods are distinguished, including the Diels–Alder “click” reaction and Schiff base formation (Hu et al., 2019). Physical cross-linking, on the other hand, relies on reversible intermolecular interactions—ionic, electrostatic, hydrophobic/hydrophilic, and hydrogen bonds. Hydrogels created using physical cross-linking are characterized by the absence of cross-linking agents, which can cause cytotoxicity, self-healing properties, injectability (at room temperature), and stimuli sensitivity (Priya et al., 2024; Bashir et al., 2020). For medical applications, the greatest attention is paid to hydrogels of natural origin. Thanks to the similarity to natural tissues, through softness and elasticity, as well as the ability to retain large amounts of water or biology solutions, it makes them present to candidates for use in regenerative medicine and tissue engineering. New possibilities offered by the modification of hydrogels or combining hydrogels with specific properties allow for the design and creation of new constructs that have the ability to respond to changing conditions that may occur in a living organism or be used for human needs (Ahmed et al., 2025) (Table 1).

**TABLE 1:** Types of natural hydrogels, their properties and forms of occurrence.

Source		Properties	Form of hydrogels
Natural hydrogels	Protein-based hydrogels	Softness	Dressings
		Toughness	Drug delivery
	Polysaccharide-based hydrogels	Biocompability	Tissue scaffolds
			Barrier materials
	Decellularized hydrogel	Stretchability	Personal care products and cosmetics
		Deformability	Sorbents for heavy metals or ionic dyes
			Agriculture agents

Hydrogels enable prolonged release of chemotherapeutic agents, thereby enhancing treatment effectiveness. Furthermore, in radiotherapy, hydrogels can serve as a carrier to load the right dose of radionuclides that can be disseminated throughout tumour cells (Norouzi et al., 2016). One of the advantages of using hydrogels and nanomaterials is reducing cancer resistance to chemotherapy (Cao et al., 2021). The development of multidrug resistance is the most common cause of death. Hydrogels enable the administration of active substances directly to the tumour (Yadav et al., 2022). Placing the chemotherapy agent in a hydrogel allows for prolonged, controlled release at a lower dose than oral or intravenous administration (Sun et al., 2023). Therefore, there is a significant need for personalized, targeted immunotherapy that allows for the local and controlled release of antibodies, cytokines, and CAR-T cells (Zhu et al., 2024). The greatest barrier to CAR-T cell therapy is ineffective infiltration of solid tumours, which is caused by physical and biological obstacles within the tumour. Hydrogels are effective drug delivery systems that enable a precise and controlled release profile of CAR-T cells (Zhou et al., 2022). Such a carrier can also act as a niche, protecting CAR-T cells from sudden loss of viability after intratumorally administration, or modify the tumour environment by neutralizing hypoxia (Shin et al., 2023). In the field of oncology, research conducted by Gollins et al. (2008) demonstrated the advantages of hydrogel dressings over gentian violet (GV) for patients undergoing radiotherapy treatment on the chest wall or in the head and neck region. However, current recommendations advise against the use of GV, as better healing outcomes are observed with the moist wound bed facilitated by hydrogel dressings. The dry crust formed over the affected area when using GV led to impaired healing (Gollins et al., 2008). Hydrogels appear to be ideal candidates for such smart wound care products (Op ’t Veld et al., 2020).

In this paper, issues related to natural hydrogels: alginates, chitosan and hyaluronic acid, peptide hydrogels, regarding their use in chemo- and radiotherapy were analysed. These biomaterials with a polysaccharide structure are characterized by biocompatibility, bioactivity, and the ability to support the regeneration of damaged tissue. Thanks to the possibility of chemical modification of these biomaterials, it is possible to create solutions with the desired features.

## 2 Alginate in oncological treatment

Alginate belongs to linear, anionic, acidic polysaccharides of natural origin. Their source is brown algae from the family *Phaeophyceae*, examples: *Ascophyllum nodosum*, *Laminaria hyperborea*, *Macrocystis pyrifera*, *Ecklonia maxima*, *Laminaria digitata*, *Laminaria japonica*, and *Scagassum*. Bacteria *Azotobacter sp*., *Pseudomonas sp*. Are also used for alginate extraction (Hasnain et al., 2020). It is composed of linear copolymers of mannuronic acid (M) and guluronic acid (G) linked by 1-4 bonds. These alduronic acid residues can form the same types of structures - MM, GG or their combinations MG, GM in different proportions. This is mainly due to the source of alginate. The arrangement of these residues affects the physical and chemical properties of alginate (Hasnain et al., 2020; Shao et al., 2025; Yin et al., 2025).

### 2.1 Alginate in radiotherapy

**FIGURE 3:**
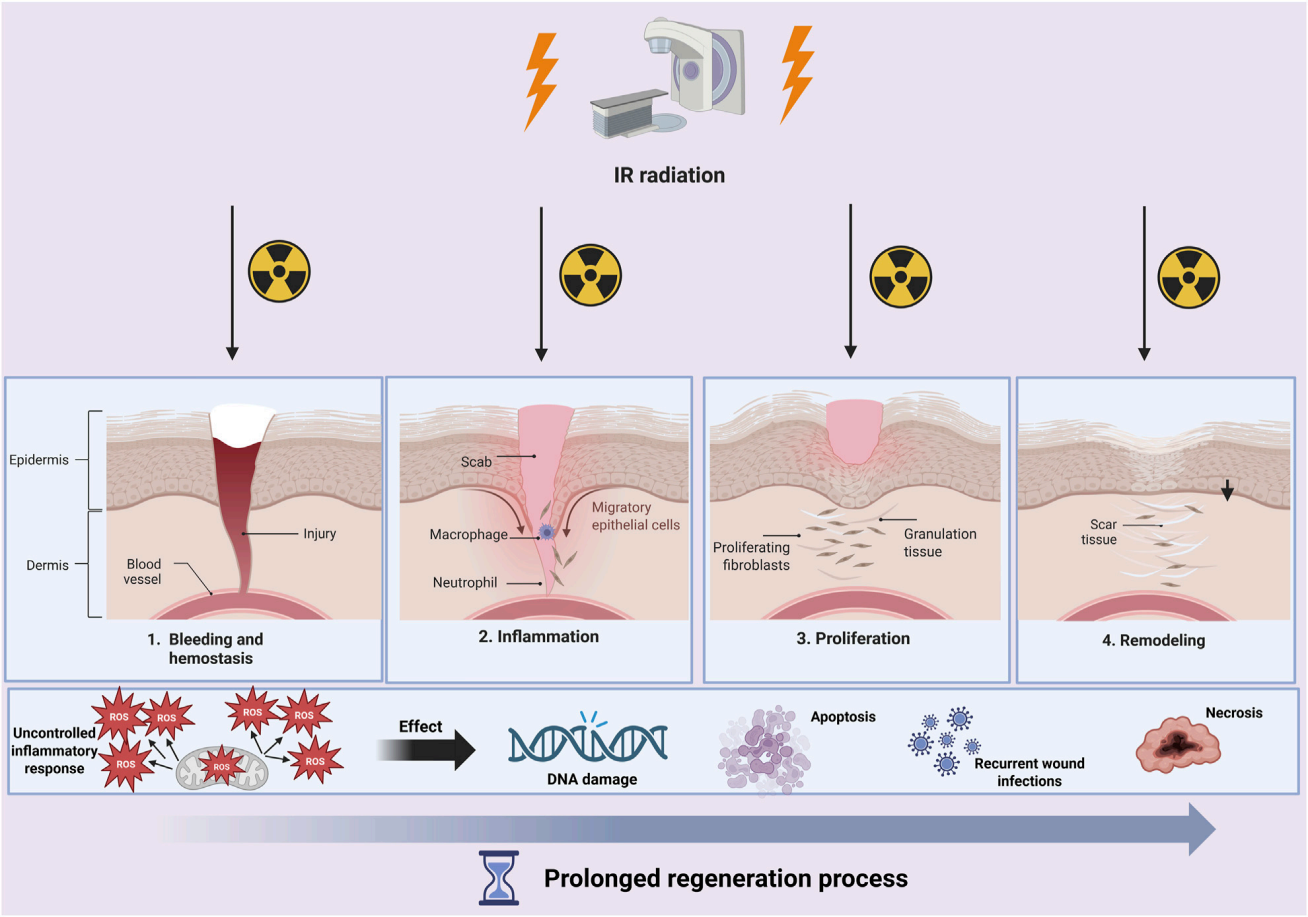
During wound healing and radiotherapy treatment, a cascade of pathological processes can occur. Not only will the healing process be prolonged, but DNA damage, cell apoptosis, or even necrosis may occur. Created with BioRender.com.

One of the key issues is that IR radiation during radiotherapy can affect the process of wound formation and healing. Wound healing during radiotherapy is more complex and complicated than in the case of typical wound healing. It is important to be aware that IR can interact with water, which constitutes 70% in the tissues of the human body, and thus produce reactive oxygen species (ROS), which are responsible for DNA damage. Overproduction of ROS leads to the development of high oxidative stress, which causes the regeneration process during healing to become longer. The presence of open wounds and a weakened immune system during the course of cancer and chemotherapy are prone to skin infections and even to cause deep tissue necrosis (Figure 3).

Simultaneously, the number of oncologic patients is increasing, in whom the administration of radiotherapy or chemotherapy can result in development of non-healing wounds. Approximately 95% of patients undergoing radiotherapy or radio chemotherapy will develop a skin condition due to the administration of high doses of ionizing radiation, known as radiodermatitis (RD) (Singh et al., 2016). The severity of radiation-induced radiodermatitis (RD) is assessed using the NCI CTCAE [*National Cancer Institute-*The Common Terminology Criteria for Adverse Events] grading scale and may manifest as erythema, dry desquamation, or moist desquamation. Discomfort, pain, and aesthetic concerns associated with this adverse effect can significantly impair patients’ quality of life, potentially leading to treatment interruptions or even discontinuation (Singh et al., 2016). Therefore, a more advanced wound healing management becomes imperative.

In advanced wound care management, Bonomo et al. (2019) highlight the pivotal role of topical calcium alginate dressings in patients experiencing radiation dermatitis (RD) due to radiotherapy and cetuximab treatment. Their findings suggest that early application of these dressings in cases of grade 2 and 3 RD with presence of moist desquamation significantly improved patients’ compliance, treatment tolerability, and reduced interruptions in radiation treatment. Therefore, authors recommend calcium alginate dressing in management of above-mentioned wounds.

The reports in Cao et al. (2024) indicate the positive effects of hydrogel and alginate dressings in a patient with grade 4 radiation dermatitis with head and neck cancer. The hydrogel dressing maintained a moist environment, supported autolytic debridement of necrotic tissue, and thus the process of phagocytosis, which was responsible for the removal of dead debris and bacteria. The alginate dressing allowed for the removal of excess exudate while providing optimal moisture. An additional advantage was the support of blood clotting and accelerated wound healing. Complete wound healing occurred within 20 days after 17 dressing changes. As the wound healed, the patient’s comfort improved significantly (Cao et al., 2024).

One example of preventing IR-induced skin injury is a dressing created by Zhang J. et al. (2021). Their hydrogel dressing, in which the main components were: alginate, hyaluronic acid, polylysine. Adding curcumin and (−)-epigallocatechin gallate to them allowed for a reduction in inflammation, due to antioxidant and anti-inflammatory properties (Zhang J. et al., 2021).

As another demonstration of the use of alginate-containing hydrogel as a dressing potentially supporting wound healing during radiotherapy is the product developed by Kodous et al. (2024). The researchers synthesized a hydrogel consisting of polyvinyl alcohol and sodium alginate, which was loaded with hesperidin. This flavonoid was subject to controlled release from the hydrogel. The hydrogel treatment reduced the expression of TNF-alpha, NFκB, iNOS and COX2 compared to the BJ-1 cell line stimulated with LPS (Kodous et al., 2024).

Due to the moisturizing properties of sodium alginate researchers (Hao et al., 2022) used it along with interferon-induced protein alpha 6 (IFI6) and graphene oxide to create a hydrogel that would find application in treating radiation-induced skin. Analyses conducted by the team of researchers on an animal model showed that such a designed hydrogel alleviates inflammation in the treatment of radiation-induced skin, and also induces granulation tissue formation, collagen deposition, or angiogenesis. Thanks to these changes, it was possible to close the wound more quickly.

In terms of treating infected wounds during oncological treatment, the Alinezhad et al. (2024) presented a hydrogel containing alginate, hyaluronic acid, polydopamine nanoparticles cross-linked with Zn^2+^. This type of dressing could potentially be used in photothermal therapy, because the use of near infrared (NIR) caused the generation of heat and the destruction of bacteria - *E. coli* and *S. aureus* (Alinezhad et al., 2024).

Alginates have found application in photodynamic therapy (Shu et al., 2021). This method is based on a phototoxic reaction, which occurs through the interaction of a photosensitising substance and light of the appropriate wavelength for the substance. The reaction occurs only in the presence of a photosensitiser, which is characterized by its ability to absorb quanta of energy, transmitted in the form of light. This causes a transition to the excited state and, in turn, a return to the ground state with the radiation of a portion of the energy in the form of the appropriate wavelength. The developed hydrogel is made resistant to luminescence through the incorporation of persistent luminescent materials and an immunoadjuvant (R837) into the Ca^2+^ alginate hydrogel, allowing for multiple loading of photodynamic cancer immunotherapy. The presented hydrogel showed high biocompatibility and allowed easy injection. The study confirmed a strong immune response and a synergistic effect of photodynamic immunotherapy to inhibit tumorigenesis (Shu et al., 2021).

A hydrogel comprising sodium alginate and catalase, labelled with the radioisotope ^131^I, was developed to alleviate hypoxia at the tumour site and destroy cancer cells using low doses of radioactivity (Chao et al., 2018). Using an injection, sodium alginate transformed into a hydrogel while binding the radioisotope and catalase at the tumour site.

Detailed information on the studies included in the text can be found in Table 2.

**TABLE 2:** List of publications discussed in the text.

Type of therapy	Type of hydrogel	Application	Method of administration	Goal of therapy	Research model	Conclusions	References
Radiotherapy	Alginate	Radiation dermatitis	Dressings	Protective factor	Human (case report)	The dressing caused re-epithelialization	Cao et al. (2024)
Radiotherapy	Alginate	IR-induced skin injury	Dressings	Protective factor	*In vivo*: female BALB/c mice	The dressing accelerated wound healing, improved vascularization, reduced inflammation and had an antibacterial effect	Zhang Z. et al. (2021)
Radiotherapy	Alginate	Chronic wounds	Application to the skin	Hesperidin-releasing carrier	*In vitro* BJ-1 human normal skin cell line	The hydrogel showed antioxidant and anti-inflammatory properties	Kodous et al. (2024)
Radiotherapy	Alginate	IR-induced skin injury	Spray	Protective factor	*In vivo*: mouse model *In vitro*: HaCaT cell line	Antibacterial, antioxidant, anti-inflammatory action	Hao et al. (2022)
Radiotherapy	Alginate	Infected full-thickness skin wounds	Injectable dressing	Protective factor	*In vitro*: Mouse embryo fibroblast cell line (NIH_3_T_3_) *In vivo*: male Sprague-Dawley rats	Antibacterial, antioxidant, hemostatic action	Alinezhad et al. (2024)
Photodynamic therapy	Alginate	-	injection into the tumour	enhancing the effect of photodynamic therapy	*In vitro*: 4T1 cells, DC2.4 Mouse Dendritic Cell Line *In vivo*: 4T1 tumor-bearing mice	hydrogel for permanent luminescence, enhanced the immunogenicity of photodynamically generated antigens through the presence of R837	Shu et al. (2021)
Radiotherapy/Chemotherapy	Alginate	solid tumors, metastatic tumors	injection into the tumour	enhancing the effect of radiotherapy	*In vivo:* *BALB/c mice, nude mice, SPF New Zealand white rabbits* *In vitro:* Murine breast cancer 4T1 cells or CT26 colorectal cancer cells, patient-derived xenograft tumour model (prostate tumour	Inhibition of tumor metastasis combined with checkpoint blockade	Chao et al. (2018)
Chemotherapy	Alginate	Lung cancer	Areosol	Drug delivery system (paclitaxel)	*In vitro*: Human non-small cell lung cancer, A549, Calu-6 cell line	*In vitro* cytotoxicity testing of paclitaxel-loaded alginate microparticles showed similar efficacy compared to free paclitaxel	Alipour et al. (2010)
Chemotherapy	Alginate	Solid tumours	Injection into the tumour	Enhancing the therapeutic response	*In vitro*: CT26 cell line, *In vivo*: 4T1-tumor-bearing BALB/c mice, BALB/c mice bearing murine CT26 colon tumours	Enabling adjuvant release synchronized with chemotherapy or radiotherapy with low individual doses, repeatedly administered over a long period of time,	Sun et al. (2021a)
Chemotherapy	Alginate	Hepatocellular carcinoma, breast cancer	Peritumoral injection,	Drug delivery system (paclitaxel, R837)	*In vitro*: human renal epithelial cells 293 T or mouse breast cancer cells 4T1 Subcutaneous - H22 murine hepatocellular carcinoma cell line Subcutaneous - 4T1 an animal model for stage IV human breast cancer *In vivo*: C57BL/6 mice	Peritumoral injection of the hydrogel induced a potent antitumor immune response against various types of cancer	Liu et al. (2024c)
Chemotherapy	Alginate	Breast cancer	-	Drug delivery system (letrozole)	*In vitro*: MCF-7 human breast cancer cell line, human foreskin fibroblast normal cell line (HFF)	The presented system demonstrated significant cytotoxicity against MCF-7 breast cancer cells. Coupling letrozole-containing niosomes to these scaffolds allowed for sustained and controlled drug release	Mahdizadeh et al. (2024)
Radiotherapy	Chitozan	Radiation skin injuries	Injectable hydrogels	Drug delivery system (Epigallocatechin gallate)	*In vitro*: Human Umbilical Vein Endothelial Cells (HUVEC) *In vivo*: Animal model (rats)	The hydrogel enhanced cell proliferation *in vitro*, scavenged ROS, and stimulated the expression of cytokines and angiogenesis-related genes. It demonstrated significant anti-inflammatory and pro-angiogenic effects in animal models	Wang et al. (2023)
Photothermal therapy	Chitozan	Tongue cancer	Injection into and around the tumour	Non-surgical therapy for tongue cancer treatment	*In vivo*: BALB/c nude mice,	Sublingual carcinoma cells were inhibited by a single PTT session	Su et al. (2021)
Radiotherapy	Chitozan	Wound skin injury caused radiotherapy	Dressings injectable hydrogel	Protective factor, drug delivery system	*In vitro*: HaCaT cell line, *In vivo*: BALB/c mice	Suppression of inflammation, regulation of macrophage polarization	Zhou et al. (2025)
Radiotherapy	Chitozan	Combined radiation-wound injury	Injectable hydrogel	Protective factor	*In vitro*: iMSCsderived from human iPSCs, L929 cell line, human umbilical vein endothelial cells (HUVECs) *In vivo*: Animal model (mice)	The hydrogel demonstrated antibacterial activity, promoting collagen deposition and angiogenesis at the wound site	Peng et al. (2024)
Radiotherapy	Chitozan	Grade IV multiforme glioblastoma	-	Attraction and accumulation of GBM cells in a hydrogel introduced into the surgical cavity after tumour resection	*In vitro*: F98 murine GBM cell line	Development of a hydrogel formulation	Parès et al. (2024)
Chemotherapy	Chitozan	-	Administration at the site of tumour resection	Drug delivery system (doxorubicin)	*In vitro*: L929 cell line, Bone-marrow-derived DCs, 4T1-Luc2 *In vivo*: female BALB/c mice	The hydrogel induced dendritic cell maturation, prevented tumor recurrence, and eliminated distal metastases	Seo et al. (2024)
Chemotherapy	Chitozan	-	-	Drug delivery system (5-fluorouracile)	-	Development of a hydrogel formulation	Wan et al. (2024)
Chemotherapy	Chitozan	Colorectal cancer	-	Drug delivery system (5-fuorouracile)	*In vitro*: HCT-116 colorectal cancer cell line	Improving the propertieIs of hydrogels	Bin Jumah et al. (2024)
Chemotherapy	Chitozan	Head and neck squamous cell carcinoma	administration at the site of tumour	Drug delivery system (Erlotinib, curcumin)	*In vitro*: FaDu cell line *In vivo*: FaDu tumour -bearing Foxn1nu mice	Optimized formulation. Anticancer efficacy	Haider et al. (2024)
Chemotherapy	Chitozan	Colon cancer	Injectable hydrogel	Drug delivery system (5-flourouracile shikonin)	*In vitro*: HUVEC, HCT-116 cell line,	Targeted drug deluvery	Daneshmehr et al. (2024)
Photodynamic therapy	Chitozan	-	-	Control photodynamic therapy activity	*In vitro*: A375 cell line, human dental pulp stem cells,	The antioxidant properties of the hydrogel. Photodynamic therapy has been proven to be selective – it acted on cancer cells	Azadikhah et al. (2021)
Chemotherapy	Chitozan	Hepatocellular carcinoma	Injectable hydrogel subcutaneously	Drug delivery system (doxorubicin)	*In vitro*: L929 cell line, HepG2 cell line,	pH-responsive hydrogels *In vitro*, they demonstrated pH-dependent gel degradation and doxorubicin release	Qu et al. (2017)
Chemotherapy/Photodynamic therapy	Chitozan	Breast cancer	Injection into the tumor	Drug delivery system (tegafur)	*In vitro*: 4T1 cell line, MCF-7 cell line, *In vivo*: 4T1 tumour-bearing BALB/c mice	Supporting the anti-cancer effect of the resulting hydrogel through the use of chemotherapy and photodynamic therapy	Zhang Z. et al. (2021)
Chemotherapy/Radiotherapy	Chitozan	Melanoma	Transdermal system	Drug delivery syste (cytostitics)	*In vitro*: L929 cell line	The physicochemical modifications used in the hydrogel enabled precise release of active substances	Kudłacik-Kramarczyk et al. (2021)
Photodynamic therapy	Chitozan	-	Injectable hydrogel	Control photodynamic therapy activity	*In vitro*: HepG2 cell line, MCF-7 cell line	Selective neutralization of cancer cells	Belali et al. (2018)
Chemotherapy/Radiotherapy	Chitozan	Oral mucositis	Nanofibrous sheet	Drug delivery (Eudragit^®^, human growth hormone)	*In vitro*: human dermal fibroblasts *In vivo*: animal model (dog)	Supporting and accelerating wound healing	Choi et al. (2016)
Radiotherapy	Hyaluronic acid	IR-induced acute skin injury	Hydrogel patch	Protective factor	*In vitro*: L929 cell line *In vivo*: Female BALB/c mice	Alleviating and accelerating the healing of radiation wounds	Xia et al. (2025)
Radiotherapy	Hyaluronic acid	Radiation-induced skin injury	Injectable hydrogel	Protective factor	*In vitro*: L929 cell line *In vivo*: C57BL/6J mice	Antioxidant properties of the hydrogel, acceleration of healing of wounds resulting from radiotherapy	Shen et al. (2025)
Radiotherapy	Hyaluronic acid	Radiation combined with skin wounds	Injectable hydrogel	Protective factor	*In vitro*: HUVECs, HaCaT cell line, L929 cell line, *In vivo*: male C57/BL/6 mice,	Antioxidant effect, improvement and acceleration of wound healing	Fang et al. (2023)
Neoadjuwant radiotherapy	Hyaluronic acid	Surgical wound after neoadjuvant radiotherapy	Wound dressing	Protective factor	*In vitro*: HUVECs, NIH3T3 cell line, *In vivo*: female BALB/c mice	Antioxidant properties, promote angiogenesis and wound healing	Wang et al. (2024)
Chemotherapy	Hyaluronic acid	Breast cancer	Intravenous administration	Drug delivery (paclitaxel)	*In vitro*: MCF-7 cell line, HL7702 cell line *In vivo*: BALB/c nude mice	Controlled release of the active substance through the hydrogel	Liu et al. (2023)
Chemotherapy	Hyaluronic acid	Solid tumors	Injectable hydrogel	Drug delivery (Endostatin)	*In vitro*: Lewis lung cancer (LLC cells), HUVECs *In vivo*: female C57 mice	Increasing the local concentration of the active substance while reducing the concentration of the active substance in serum	Wang et al. (2021)
Chemotherapy	Hyaluronic acid	Breast cancer	Intravenous administration	Drug delivery (cytochrome c)	*In vitro*: MCF-7 cell line, U87 cell line, *In vivo*: MCF-7 breast tumour xenografts (female nude mice)	Targeted administration of the active substance and its release at the site of cancer cells, causing a strong anti-cancer effect	Li et al. (2016)
Radiotherapy	Hyaluronic acid	Osteoradionecrosis	Administration after extraction or after the formation of osteoradionecrosis	Drug delivery (rat mesenchymal stem cells, BMP-2)	*In vitro*: rat mesenchymal stem cells *In vivo*: male Spraguee-Dawley rats	Accelerated bone healing following BMP-2 administration. Administration of BMP-2 and stem cells allowed for the effective treatment of osteoradionecrosis	Jin et al. (2015)
Radiotherapy	Hyaluronic acid	Lung cancer	Injectable hydrogel	Drug delivery (cytarabine)	*In vitro*: Lewis lung cancer cell line *In vivo*: LLC- tumour-bearing female C57BL/6J mice	Combining the hydrogel with radiotherapy allowed for inhibition of lung tumor growth in a mouse model	Tang et al. (2019)
Radiotherapy	Peptide hydrogel	Radiation-induced skin injury	Application to the skin	Induction of angiogenesis	*In vitro*: 3T3 cell line, HUVECs, *In vivo*: female BALB/c mice	Antioxidant and anti-inflammatory effects	Hao et al. (2023)
Radiotherapy	Peptide hydrogel	Radiation-induced ototoxicity	Injectable hydrogel	Delivery system (dexamethasone)	*In vitro*: HEI-OC1 cell line HNE-1 cell line, CNE-2 cell line, HSC-3 cell line CAL-27 cell line	Controlled and sustained release of the active ingredient demonstrated anti-inflammatory properties. Intratympanic injections of the hydrogel protected hair cells	Liu et al. (2024b)
Chemotherapy	Peptide hydrogel	Osteosarcoma	Injectable hydrogel	Delivery system (doxorubicin)	*In vitro*: murine K12 osteosarcoma cell line, NIH3T3 fibroblast cell line, *In vivo*: female BALB/c mice with K12 cells,	An increased accumulation of the active substance in tumors was demonstrated	Zhu et al. (2023)
Chemotherapy	Peptide hydrogel	Esophageal cancer	Injectable hydrogel (intrtumorally)	Delivery system (doxorubicin)	*In vitro*: AKR oesophageal cancer cell line, HEEC human normal esophageal epithelial cell line, *In vivo*: NOD/SCID mice	By extending the duration of administration of the active substance, it reduced systemic toxicity	Ye et al. (2024)
Chemotherapy	Peptide hydrogel	Melanoma	Injectable hydrogel	Delivery system (doxorubicin)	*In vitro*: B16-F10 cell line, Bone marrow-derived dendritic cells (BMDCs) harvested from femurs and tibiae mice, RBCs isolated from fresh mouse blood, *In vivo*: Female C57BL/6 mice	Thanks to the controlled release of the active substance, a strong anti-cancer effect was observed	Jin et al. (2018)
Chemotherapy	Peptide hydrogel	-	Injectable hydrogel (intratumorally)	Delivery system (paclitaxel)	*In vitro*: human HCC cell line (HepG2), murine H22 hepatoma cell line, HUVECs, *In vivo*: female BALB/c mice	Increasing the accumulation of the active substance at the tumor site	Raza et al. (2019)
Chemotherapy	Peptide hydrogel	-	Injectable hydrogel (areas of cancerous tissue)	Delivery system (gemcitabine, paclitaxel)	*In vitro*: 4T1 cell line, *In vivo*: BALB/c female mice (4T1- tumo- bearing mice)	Both active substances ensured a long-lasting and concentrated release	Liu et al. (2022)

**TABLE 3:** List of clinical trials using natural hydrogels with status: completed (clinicaltrials.gov from 02/09/2025).

Type of biomaterial	Disease entity	Name of the study	NTC number	Phase
Alginate	Hydrogel Injection to Assist Endoscopic Submucosal Dissection	Hydrogel Injection to Assist Endoscopic Submucosal Dissection	NCT03321396	Not Applicable
Hyaluronic acid	Actinic Keratoses	Effects of Topical Diclofenac on Tumor Metabolism	NCT01935531	IV
Hyaluronic acid	Cancer Pain	35kDa Hyaluronan Fragment Treatment of Colorectal and Rectal Cancer	NCT06209970	Not Applicable
Hyaluronic acid	Ototoxicity	Study to Evaluate Safety and Efficacy of DB-020 to Protect Hearing in Patients Receiving Cisplatin for Cancer Treatment	NCT04262336	I
Hyaluronic acid	Genitourinary Syndrome of Menopause	Management of Cancer Therapy Related Vulvovaginal Atrophy	NCT05782920	Not Applicable
Hyaluronic acid	Prostatic Adenocarcinoma	Hypofractionated Radiotherapy Versus Stereotactic Irradiation With Hyaluronic Acid	NCT02361515	Not Applicable
Hyaluronic acid	Non-Invasive Papillary Carcinoma of Bladder	ONCOFID-P-B in the Intravescical Therapy of Patients With Non-muscle Invasive Cancer of the Bladder.	NCT04661826	II
Hyaluronic acid	Recurrent Prostate Cancer, Brachytherapy Remedial,	Brachytherapy for Recurrent Prostate Cancer	NCT01956058	II
Hyaluronic acid	Breast Cancer Endometrial Cancer	Non-Hormonal Vaginal Moisturizer in Hormone-Receptor Positive Postmenopausal Cancer Survivors Experiencing Estrogen Deprivation Symptoms on Vulvovaginal Health	NCT01738152	Not Applicable
Hyaluronic acid	Advanced or Metastatic Solid Tumors Advanced or Metastatic Colorectal Cancer (mCRC)	First-in-human Study of CA102N Monotherapy and CA102N Combined With Trifluridine/Tipiracil (LONSURF) in Subjects With Advanced Solid Tumors	NCT03616574	I

### 2.2 Alginate in chemotherapy

In oncological treatment, alginate microparticles combined with paclitaxel have been used as a carrier of cytostatic drugs (Alipour et al., 2010). Researchers used calcium alginate as a mucoadhesive polymer, and its microparticles stick to the mucosa for a long time and are therefore used as vehicles for specific drug delivery to mucosal tissues. The conducted *in vitro* studies showed that exposure of cells to the paclitaxel alginian carrier and pure paclitaxel inhibited cell growth in a similar way, which depended on concentration and time.

The innovative use of alginate in another study resulted in the release of immunoadjuvants during multiple sessions of chemotherapy/radiotherapy (Sun L. et al., 2021). The component of this hydrogel was sodium alginate, which was conjugated to an ATP-specific aptamer that simultaneously hybridizes to the immunoadjuvant CpG oligonucleotide. After injection, an alginate hydrogel is formed at the tumour site. Its premise was that low doses of oxaliplatin or X-rays would induce immunogenic tumour cell death, which would trigger the release of ATP, which would bind to the ATP-specific aptamer, and this would lead to the release of CpG.

Using the properties of alginate as a carrier, which has the ability to control the release of substances contained in it, Liu Q. et al. (2024) created a type of “*in situ* vaccine.” The researchers injected a hydrogel that formed near the tumour. It allowed the controlled release of nanoparticle paclitaxel bound to albumin, which shows better efficiency than paclitaxel itself and does not cause allergic conditions, and can also be enriched at the tumour site. Additionally, the hydrogel was attached to the immunostimulant agent R837 – Imiquimod, a TLR9 receptor agonist that promotes dendritic cell maturation, as well as migration, antigen presentation and induction of tumour-specific T lymphocytes. Elements of the immune system such as chemokines and receptors were also significantly involved. Injecting alginate near the tumour allows for a reduced dose of the drug and fewer injections, which reduces toxicity to healthy cells and tissue (Liu Q. et al., 2024).

Carriers for chemotherapeutic drugs can be created using proven and innovative 3D printing technology. Mahdizadeh et al. (2024) used niosome technology, nanocarriers consisting of cholesterol-based vesicles and non-ionic surfactants forming bilayer structures. Their main advantage is low toxicity due to the non-ionic properties of the surfactants (Mahdizadeh et al., 2024).

Detailed information on the studies included in the text can be found in Table 2.

## 3 Chitosan in oncological treatment

Chitosan is a linear polysaccharide that occurs naturally in the shells of crustaceans, insects and the cell walls of some fungi. It is obtained in the process of deacetylation of chitin. As a biomaterial, it has antibacterial, antifungal and gelling properties (Tymińska et al., 2024). Due to its cationic nature, it is used with negatively charged groups found in proteins, enzymes and other polymers (Li et al., 2020; Sharkawy et al., 2020).

Chitosan, due to its mechanical properties, has been used in the treatment of wounds exposed to radiation as a dressing in the form of a film. In combination with keratin, which has the properties of rapid hemostasis, peripheral nerve repair and supports wound healing, but poor mechanical properties, it resulted in the creation of a product that is easy to use and has high mechanical strength. Studies on a rat model showed an improved healing marker, and a biopsy of the wound site after 7 and 14 days confirmed increased angiogenesis and collagen production (Wang et al., 2025).

### 3.1 Chitosan in radiotherapy

The current treatment of wounds resulting from radiotherapy involves biological agents such as the administration of epidermal growth factor, synthetic drugs, including corticosteroids, statins, and vitamins. Their use is expensive for the patient and is burdened with side effects. This type of therapy is not precise. Wang et al. (2023) proposed a type of injectable hydrogel based on chitosan and gelatin, among others, to which epigallocatechin gallate, which captures hydroxyl radicals, was added. In cell studies and those conducted on an animal model, the hydrogel constructed in this way demonstrated an angiogenesis-promoting and anti-inflammatory effect (Wang et al., 2023).

**FIGURE 4:**
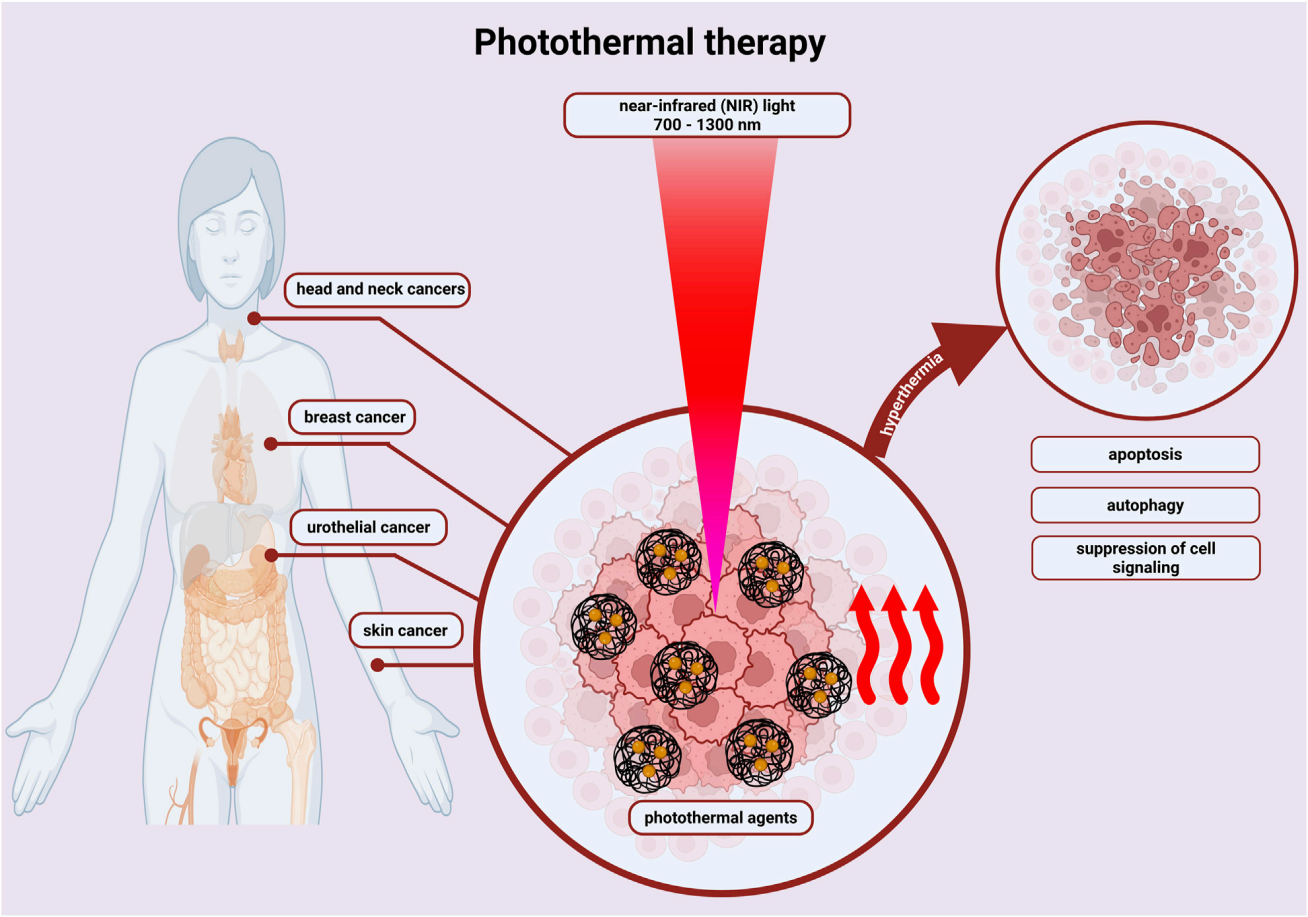
By irradiating photothermal agents with near infrared (NIR) light, hyperthermia is induced, which destroys cancer cells through energy transfer - increasing temperature. Photothermal therapy activates apoptosis, autophagy or suppresses cell signalling, inducing cancer cell death. This is done with a shorter treatment time, reduced pain and fewer side effects. Created with BioRender.com.

In the field of oncology, Su et al. (2021) developed hydrogel, which is near-infrared light-responsive and applicable to peri-tumour injection. This could be used for photothermal therapy, particularly in tongue tumours. Photothermal therapy involves the use of compounds, photothermal agents and local heating of the tissue (Figure 4). The photothermal agent absorbs the laser light, resulting in the excitation of electrons and their transition from the ground state to a higher level. The energy thus supplied is given off in the form of heat instead of by photon emission. This method is characterized by high efficiency and stability, and high stability in time and space. Su et al. used biomaterial, which had a good biocompatibility and strong photothermal effect because of the formed network by negatively charged proteins, chitosan molecules and Ag_3_AuS_2_ nanoparticles. The researchers, through the mouse model of tongue cancer used, demonstrated that sublingual cancer cells were eliminated during a single photothermal therapy. The hydrogel system used, thanks to its low immunogenicity, reduced direct biotoxicity and improved therapeutic effects. One of the unique properties of chitosan is that it is a linear polymer with a positive charge. Combination with negatively charged carboxymethylcellulose results in the formation of a polyelectrolyte complex, in which the obtained hydrogel controls the release of substances placed in it. Zhou et al. (2025) presented a hydrogel based on these substrates with the addition of cannabidiol, the effect of which reduces oxidative stress. Studies conducted on mice confirmed and an increase in collagen synthesis.

The addition of exosomes, which have properties that promote angiogenesis in ischemic tissues and improve the activity of keratinocytes, fibroblasts, endothelial cells and components of the immune system, and their introduction into a hydrogel may bring the desired effects in the case of treatment of radiation injuries. The team of Peng et al. (2024) undertook this task and created a hydrogel based on quaternized chitosan and oxidized sodium alginate with exosomes enclosed in them, which was applied superficially to the wound to obtain a protective barrier. The synthesized hydrogel showed protective properties against microorganisms, promoted rapid re-epithelialization, angiogenesis and collagen deposition (Peng et al., 2024).

The team of Parès et al. (2024) presented a different point of view regarding chitosan hydrogel. Their area of interest was glioblastoma multiforme stage IV. In this aggressive form of cancer, healthy cells surrounding brain tissue are attacked - in them, cancer cells multiply. New reports speak of the attraction and accumulation of glioblastoma multiforme cells in a hydrogel introduced to the site of tumour resection, and then a high dose of radiotherapy is administered. The researchers prepared a macroporous hydrogel consisting of chitosan, sodium alginate, cross-linked with genipin and calcium chloride. Studies conducted on the F98 mCherry cell model distributed, accumulated and were retained throughout the gel volume (Parès et al., 2024).

Detailed information on the studies included in the text can be found in Table 2.

### 3.2 Chitosan in chemotherapy

Chitosan hydrogels may provide a drug delivery platform for altering antitumor immunity to enhance immunotherapy. Seo et al. (2024) developed a hydrogel based on methacrylated chitosan glycol and a complex of DNA-a sodium salt extracted from salmon testes and doxorubicin. With this combination, controlled release of the DNA/DOX complex was proven to protect against tumour recurrence and metastasis and induce an antitumor response (Seo et al., 2024).

5-Fluorouracil is the most commonly prescribed drug in the treatment of solid tumours. Due to its rapid metabolism by dihydropyrimidine dehydrogenase and short half-life, limited bioavailability and high systemic toxicity, new methods are sought to target it and improve therapeutic efficacy. Chitosan as a nanocarrier has the advantage of creating a uniform particle size distribution, and the release of the substance contained in it is pH dependent. It is important to know that the pH range occurring in tumour tissues differ from the pH range in healthy tissue. Researchers have developed various methods of cross-linking chitosan so that it optimally releases the chemotherapeutic agent contained in it (Wan et al., 2024).

Reducing the side effects of chemotherapeutics is a primary concern in the design of modern drug delivery systems. Bin Jumah et al. (2024) designed a biocomposite with improved physicochemical and biological properties, consisting of a zinc-phosphate/hydroxyapatite hybrid form in core-shell nanostructure and functionalized with both chitosan and β-cyclodextrin as a 5-fluorouracil carrier. Studies on the colon cancer cell line confirmed the antitumor activity and the controlled release profile of 5-fluorouracil (Bin Jumah et al., 2024).

The idea of creating an injectable drug delivery nanosystem that would allow for better bioavailability of the target drug used in chemotherapy allowed the development of the concept by the team of Haider et al. (2024). Erlotinib is ideally suited for this type of system because it has difficulties in dissolving in water, gastrointestinal absorption and low bioavailability. *In vivo* studies have confirmed that intratumorally injection of the preparation causes a slowdown in tumour growth (Haider et al., 2024).

Similar conclusions were used by Daneshmehr et al. (2024), who studied the attachment of 5-fluorouracil and schionine to hydrogel nanoparticles of chitosan and pectin. They were prepared by two methods: mixing and coating. The studies carried out on cell lines for the treatment of colon cancer indicated that the mixing method showed the appropriate loading power (Daneshmehr et al., 2024).

**FIGURE 5:**
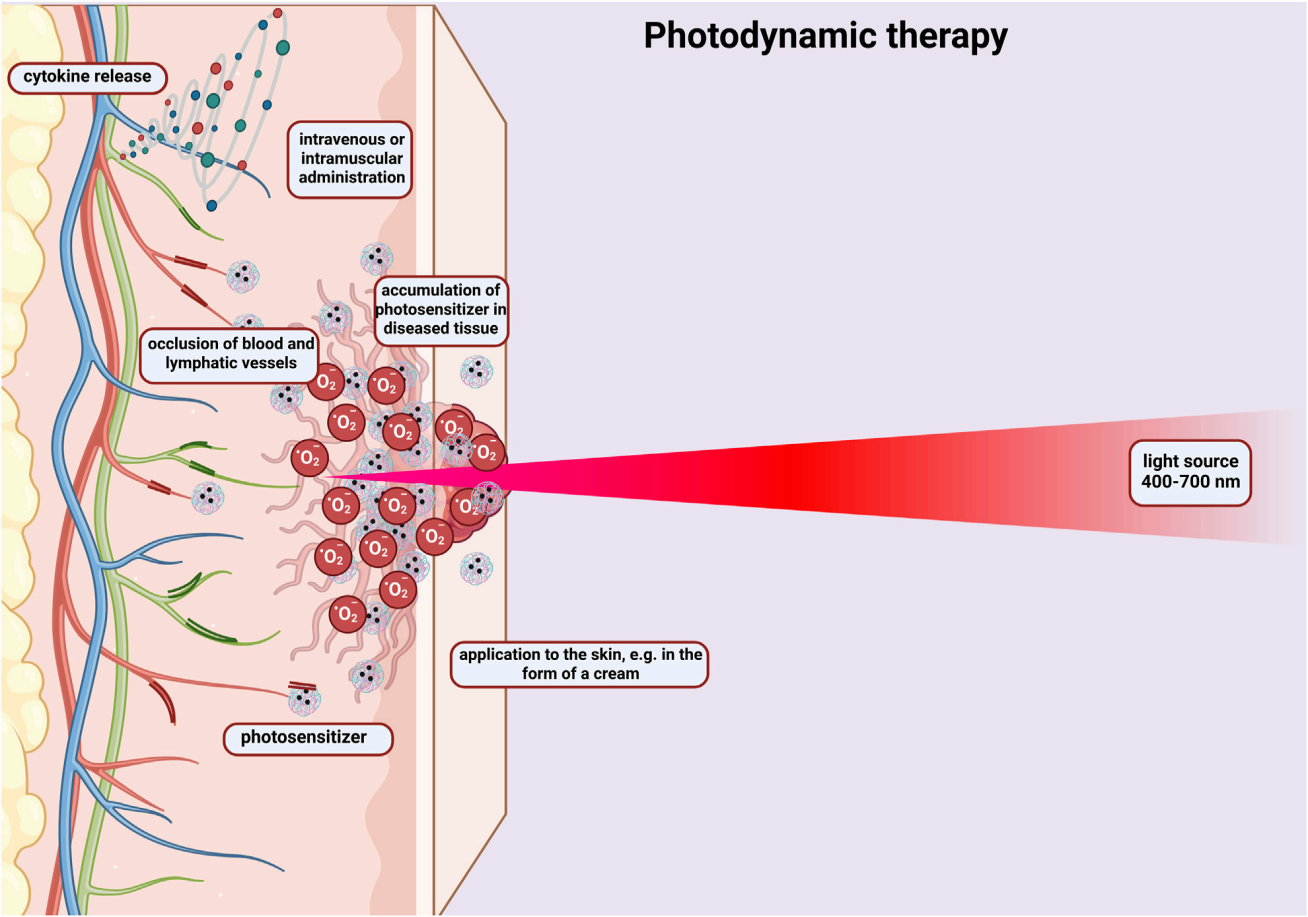
Diagram showing the principle of photodynamic therapy. Activation of a photosensitizer administered intravenously or to the skin in the form of a cream with visible light, which leads to the formation of free oxygen radicals that destroy cancer cells. The photosensitizer penetrates the cells changed by the cancer. The therapy causes occlusion of blood and lymphatic vessels and the release of cytokines. Created with BioRender.com.

Hydrogel based on chitosan and tannic acid was created as an antioxidant crosslinker whose goal was to control the activity of photodynamic therapy used in the treatment of cancer (Azadikhah et al., 2021). In this method, it should be mentioned that photosensitizers cause the formation of reactive oxygen species during photochemical reactions. This results in oxidative damage to cancer cells (Figure 5). The research showed that chitosan with the addition of tannic acid formed a three-dimensional structure that had good mechanical strength and antioxidant activity. The designed hydrogel effectively inhibited tumour growth and protected the dental pulp stem cells from phototoxicity.

Newly developed hydrogels containing N-carboxyethylchitosan has a pH-sensitive property, which were presented as carriers for doxorubicin (Qu et al., 2017). Analyses showed that the pH-sensitive hydrogels released more doxorubicin around/inside the tumour than in normal healthy tissue. With such carriers, it is possible to have a more effective, targeted delivery site for the anti-tumour drug, reduce side effects and protect healthy tissue not occupied by the tumour.

What’s more a biomimetic, thermosensitive hydrogel had ability to incorporate heterodimers of tegafur, protoporphyrin IX (Zhang Z. et al., 2021). The action was to combine chemotherapy with photodynamic therapy. Due to the laser effect, the concentration of reactive oxygen species increased in the tumour, and this phenomenon was important for the release of the drug. The drug injected, and released only with the laser action, reduced the disorderly effects. The researchers opted for chitosan and silk sericin. The thermosensitive properties exhibited by chitosan played an essential role in the choice of carrier.

A related case of the use of chitosan in oncology is chitosan-based hydrogel that contained albumin beads, which as a carrier are characterized by accumulation near cancer cells, and aloe vera juice, which is characterized by antibacterial and anti-inflammatory properties (Kudłacik-Kramarczyk et al., 2021). The hydrogel constructed in this way was to be used for the treatment of skin cancer or burn wounds resulting from radiation therapy. Analyses showed that the tested hydrogels were non-toxic to L929 mouse fibroblasts, and albumin beads, which are a component of the hydrogel.

A hydrogel incorporating chitosan and conjugated with porphyrins was developed to enhance the application of photodynamic therapy (Belali et al., 2018). The hydrogel designed in this way caused the excitation energy to be transferred to the porphyrin unit, and this resulted in an improved release of singlet oxygen. Cytotoxicity and phototoxicity studies of chitosan-based hydrogels highlighted the property to selectively kill cancer cells and protect healthy body cells.

Patients undergoing oncological treatment face various side effects of this therapy, one of them is ulceration of the mucous membranes. Choi et al. (2016) prepared two-layer electrospun fiber sheets that consisted of Eudraugite, chitosan and human growth hormone. Fiber sheets containing growth hormone resulted in better proliferation of human fibroblasts. Studies on an animal model proved that this type of dressing could improve the healing of mucosal ulcers.

Detailed information on the studies included in the text can be found in Table 2.

## 4 Hyaluronic acid in oncological treatment

Hyaluronic acid (HA) is a linear mucopolysaccharide composed of repeating units of β-1,4-D-glucuronic acid and β-1,3-N-acetylglucosamine. The alternating β-1,3 bonds are responsible for the high elasticity and solubility of hyaluronic acid polymers. It is the main component of the extracellular matrix (Gupta et al., 2019; Buckley et al., 2022; Valachová et al., 2024; Encyclopedia, 2025). HA has some disadvantages, it plays a role in tumour physiology, increased hyaluronan level mostly is associated with poor prognosis. HA and hyaluronidase have both: pro- and antitumor effect, which is concentration and origin dependent (Salwowska et al., 2016). HA has also poor mechanical properties and rapid degradation *in vivo,* but chemical modification and crosslinking can overcome disadvantages (Chircov et al., 2018).

### 4.1 Hyaluronic acid in radiotherapy

An example of creating hydrogel dressings using HA is the work of Xia et al. (2025), in which they presented a dressing that protected against radiation and at the same time had antioxidant properties. Additionally, the structure of the patch contained epidermal growth factor (EGF), which supported skin healing. The dressing designed by the researchers uses the property of releasing large amounts of free radicals, then the bonds in the structure of the dressing can be oxidized and transformed into a hydrophilic sulfone. The consequence of this action is the stretching of the hydrogel network and the release of the encapsulated EGF (Xia et al., 2025).

The use of HA cream in patients experiencing radiation dermatitis (RD) shows promise in alleviating symptoms. Preliminary data presented by Gracy et al. (2004) suggest that topical application of HA protected cultured fibroblasts from radiation damage. Chan et al. (2014) advocate for the additional use of HA to provide symptomatic relief, among other topical agents, as *in vivo* studies have not yet definitively established its benefits in preventing the development of RD.

Another example of using a scheme in which the primary element is the elimination of free radicals formed in wounds during radiotherapy is the creation by Shen et al. (2025) of an injectable hydrogel with Pluronic F127 diacrylate and hyaluronic acid methacryloyl, and nanoparticles of Prussian blue and resveratrol placed in them. It was proven that the hydrogel promoted the migration of fibroblasts to the site of oxidative stress A subsequent exampleis placing proteins and stem cells in hydrogels. Jin et al. (2015) created a hyaluronic acid-based hydrogel loaded with rat mesenchymal stem cells and bone morphogenetic protein-2 (BMP-2). The biomaterial was designed to affect bone healing in osteoradionecrosis of the rat mandible. Anchoring the hydrogel with stem cells and BMP-2 increased bone mineral density and bone volume.

An important aspect is the inhibition of the growth of cancer cells. Tang et al. (2019) developed a hydrogel that included hyaluronic acid, cytarabine and tyramine in its formulation. Combination therapy with radiotherapy significantly reduced 18F-FDG uptake, triggered increased apoptosis and histone H2AX phosphorylation, cell cycle arrest in the G2/M phase, and reduced the proliferation rate in tumour cells compared to monotherapy.

To mitigate the damage caused by radiotherapy, Fang et al. (2023) designed a hydrogel that included cross-linked carbohydrazide-modified gelatin and oxidized HA, polydopamine nanoparticles, and extracellular vesicles secreted by mesenchymal stem cells. *In vitro* studies showed enhanced cell viability, as evidenced by stimulation of proliferation and migration. Proteomic studies proved that the hydrogel alleviated the radiation-related wound microenvironment by regulating adipocyte and hypoxia-related pathways.

The advantage of hyaluronic acid as a biomaterial is that it can combine with collagen and fibronectin, i.e., matrix structures in tissues, creating a temporary scaffold. Such an organization of materials can effectively promote cell adhesion, migration and proliferation, collagen synthesis. However, its disadvantages are also noticed: poor durability in an aqueous environment, sensitivity to hyaluronidase or free radicals. Therefore, Wang et al. (2024) created a hydrogel based on hyaluronic acid and a self-assembling peptide with attached cordycipin. Hyaluronic acid was to strengthen non-covalent interactions with the peptide. The scaffold skeleton created in this way supported cell adhesion and proliferation.

Detailed information on the studies included in the text can be found in Table 2.

### 4.2 Hyaluronic acid in chemotherapy

Hyaluronic acid has high affinity for the CD44 receptor, which is overexpressed in cancer cells. Liu et al. (2023) used this property and designed micelles in which modified hyaluronic acid served as a carrier to bind to CD44. *In vivo* and *in vitro* studies confirmed inhibition of tumour growth. And the drug delivery vehicle itself was nontoxic.

A very valuable technological achievement is the placement of drugs in hydrogel carriers. In studies by Wang et al. (2021), HA-tyramine was utilized to create a carrier for endostatin, which could be locally injected. The hydrogel prepared in this way showed longer half-life, less systemic toxic side effects. ES/HA-Tyr revealed better anti-angiogenic effect tested on cellular model and anti-tumour effect with radiotherapy tested on animal model. Intra-tumour administration allowed the drug to increase its concentration locally, thereby reducing serum endostatin levels.

Similarly, Li et al. (2016) developed redox-sensitive and intrinsically fluorescent photoclick hyaluronic acid nanogels to deliver cytochrome c to xenografted MCF-7 breast tumours in mice. The study confirmed that the production of the hyaluronic acid nanogel had high specificity. The prepared nanogel allows rapid targeting of tumour cells and rapid release of therapeutic proteins under cytoplasmic reduction conditions while providing potent anti-tumour activity. By exhibiting intrinsic fluorescence, potential use for *in vivo* tumour monitoring is possible.

Detailed information on the studies included in the text can be found in Table 2.

## 5 Peptide hydrogels in oncological treatment

Peptide hydrogels are a soft material platform that uses amino acids and peptides as building blocks that can retain water or transform into a hydrogel under physiological conditions. Peptides enable the formation of hydrogels and nanoparticles through self-assembly, which is formed similarly to the beta-arc and alpha-helical structures in peptide networks and globular protein structures (Fu et al., 2021; Mitrovic et al., 2023; Nizam et al., 2025).

### 5.1 Peptide hydrogels in radiotherapy

Skin changes after radiotherapy are a serious problem that occurs in patients undergoing this therapy. Hao et al. (2023) created a hydrogel with antioxidant properties, which included a heparinomimetic peptide. The created system was intended to be used in the process of repairing skin damage caused by radiation. The researchers used the K16 peptide, which had the ability to self-assemble into a hydrogel that formed a 3D mesh and thus created an environment resembling the extracellular matrix. Studies showed that this peptide also supported cell proliferation, migration, angiogenesis, well as protecting cell DNA against radiation-induced damage. The hydrogel dressing prepared in this way promoted collagen deposition and inhibited early wound degradation.

Researchers have also drawn attention to the problem of radiation-induced ototoxicity. This occurs in head and neck cancers. The mechanism of this phenomenon is not fully understood, probably radiolysis of water, which is induced by radiation, is the hotspot of cochlear cell destruction. Liu J. et al. (2024) proposed an injectable peptide hydrogel based on RADA with attached dexamethasone. Additionally, the developed peptide hydrogel reactivated the mTOR signalling pathway, which allowed protection of neuronal cells. During radiotherapy, this signalling pathway is suppressed and neuronal cells in the cochlea are destroyed.

The property of peptide hydrogels is to prolong drug retention by spatial restriction. Zhu et al. (2023) constructed a nonapeptide hydrogel with doxorubicin that responds to changing pH in the environment. The developed nonapeptide called P1 belongs to the type of self-assembling peptides, similar to surfactants. The proposed model had high efficiency of doxorubicin encapsulation, retained sensitivity in an acidic environment. Detailed information on the studies included in the text can be found in Table 2.

### 5.2 Peptide hydrogels in chemotherapy

The team of Ye et al. (2024) had a similar assumption. In their work, they presented the IEIIIK peptide, which had the ability to surround doxorubicin and self-assemble into a hydrogel under physiological conditions, and after injection into the acidic environment of the tumour, was able to release doxorubicin and allowed its accumulation within the tumour cells.

An important challenge is to create effective peptide hydrogels as carriers of cytostatic drugs, which, thanks to their properties, can influence the change of the microenvironment inside the tumour. Jin et al. (2018) created a of melittin-RADA_32_ hybrid peptide hydrogel, which was loaded with doxorubicin. It was designed to affect the immunosuppressive tumour microenvironments in melanoma.

This biomaterial has been shown to have the property of controlled drug release and to affect immune cells. As for subcutaneous and metastatic tumours, this hydrogel showed strong anti-tumour efficacy in their case. Raza et al. (2019) developed a FER-8 peptide hydrogel loaded with paclitaxel, which was also pH-sensitive. Analyses in an animal model showed that the peptide hydrogel, as a carrier for paclitaxel, significantly increased the amount of drug in tumour tissues, and by direct injection into the tumour, showed prolonged retention at the injection site. Due to the drug’s release property in the presence of acidic pH, prolonged delivery of the drug and thus increased tumour inhibition was possible.

In an attempt to overcome the reduced efficacy when administering combination therapy, when two or more drugs are used that would reach the target at the same time, Liu et al. (2022) were designing carriers that could release the active substances at the same time. The study showed that the peptide carrier for both drugs, when injected into the tumour site, results in a prolonged and concentrated release -gemcitabine, which is hydrophilic in nature, is released in large amounts, while paclitaxel, which is hydrophobic in nature, is released slowly, allowing for prolonged drug action and increased efficacy.

Detailed information on the studies included in the text can be found in Table 2.

## 6 Limitations of hydrogels in oncology therapies

Although hydrogels are a promising strategy for therapeutic applications in oncology, they also have certain limitations (Omidian et al., 2024). The first is the detailed replication of the mechanical properties of natural tissues. In practice, tissues are characterized by significantly more complex mechanics, and simple parameters such as Young’s modulus are not sufficient to achieve a biomimetic environment (Roth et al., 2023; Fatima and Almeida, 2024). Another aspect to consider when designing hydrogels is the excessively rapid degradation of the carrier (Zhu et al., 2025). This can result in a lack of control over the release of the active substance, thus leading to a failure to achieve the intended therapeutic effects (Segneanu et al., 2025). There is also the possibility of uneven distribution of the hydrogel at the site of administration, which can lead to unpredictable body reactions (Almawash et al., 2022). Technological limitations are another obstacle to the use of hydrogels in oncological therapies. The challenge is the reproducible synthesis of hydrogels with specific mechanical and biological properties. It should be noted that there is little data on the long-term effects of hydrogels in oncological therapies. Most reports and publications refer to studies on animals and cell lines. Translating these findings into the human body could yield completely different results. This could result in the termination of promising studies. It should be noted that there is little data on the long-term effects of using hydrogels in cancer therapies. One important factor is financial considerations. Developing high-quality hydrogels on a large scale is expensive. Clinical registration and eventual commercialization of the product are a separate issue. The process can take several years and involve significant costs. Various regulatory aspects, including legal and scientific requirements, must also be considered (Almawash et al., 2022; Roth et al., 2023; Fatima and Almeida, 2024; Omidian et al., 2024).

## 7 Future perspectives

Hydrogels represent a highly promising class of materials for clinical applications. Future research is expected to focus on the development of intelligent, stimuli-responsive systems (e.g., triggered by enzymes, pH or light/laser stimulation), enabling controlled and localized drug release within tumor tissues. Another important direction involves exploring the role of hydrogels in inhibiting metastatic spread by serving as anti-adhesion and protective barriers. Hybrid hydrogels incorporating functional particles such as liposomes or exosomes, hold great potential for enhancing the targeted delivery of therapeutic agents. Moreover, the integration of mRNA into hydrogel systems opens opportunities for advancing personalized cancer therapies. Efforts should also be directed toward the design of fully biodegradable and immunologically safe hydrogels to ensure clinical safety and efficacy. Finally the incorporation of artificial intelligence, and 3D printing technologies into hydrogel development is likely to accelerate the creation of patient-specific solutions, advancing both regenerative medicine and oncology.

## 8 Conclusion

Hydrogels based on natural components such as alginate, chitosan, hyaluronic acid or self-assembling peptide hydrogels show great potential in oncological treatment. Thanks to their unique properties such as biocompatibility and biodegradability, the ability to absorb large amounts of water and create 3D structures, they are becoming modern, desirable solutions in the field of drug carriers and dressings (Zielińska et al., 2023; Satchanska et al., 2024; Arif, 2025). Translating the results and innovative solutions from basic research into clinical trials is very tedious. This is evidenced by the number of clinical trials using hydrogels in oncology therapies that have been completed. Only two types of hydrogels have been used in research: alginate and hyaluronic acid. The patient’s wellbeing must be taken into account, because the body is in a weaker condition due to the fight against cancer, additional side effects such as the appearance of wounds and ulcers, and consequently disfigurement, negatively affect the further treatment process. The formation of radiation-induced wounds causes the accumulation of ROS (Liu C. et al., 2024). This phenomenon disrupts the redox balance and triggers an inflammatory response, thus increasing the production of proinflammatory cytokines and activating signalling pathways that lead to cell damage (Pizzino et al., 2017). This causes cell cycle arrest and the production of aging-related factors. Hydrogels and their modifications can act as free radical scavengers and reduce oxidative damage in healthy tissues (Joorabloo and Liu, 2024). The cited studies confirm the healing-promoting properties in the treatment of wounds during radiotherapy treatment. Drugs used in chemotherapy have low specificity, limited solubility and bioavailability, and when taken in large doses, they have a toxic effect on the body (Zhang et al., 2022; Prieložná et al., 2024). It has been proven that hydrogels as drug carriers cause prolonged drug retention at the tumour site, while limiting diffusion to surrounding tissues (Ma et al., 2022; Mikhail et al., 2023; Lu et al., 2024). This action allows for minimizing systemic toxicity and drug access even to poorly vascularized tumour areas.

The last two decades have seen significant advances beyond oncology therapies, including surgical techniques for tumor resection. A milestone was the introduction of robotics (Chatterjee et al., 2024) and the use of techniques to assess tissue perfusion using indocyanine green (Cassinotti et al., 2023). However, the progress that has been made has not significantly changed the problem of leakage in the digestive tract, which is 3%–30% (Chiarello et al., 2022). Research is currently underway into the use of hydrogels as an adjunct method in gastrointestinal anastomoses, although their use is currently experimental. The additional use of hydrogels would benefit from reducing the rate of leaks at the anastomosis site. However, one of the drawbacks of using hydrogels is the difficulty in maintaining this type of preparation at the anastomosis site, which should be applied externally to the anastomosis, where the outer layer is smooth serosa.

**FIGURE 6:**
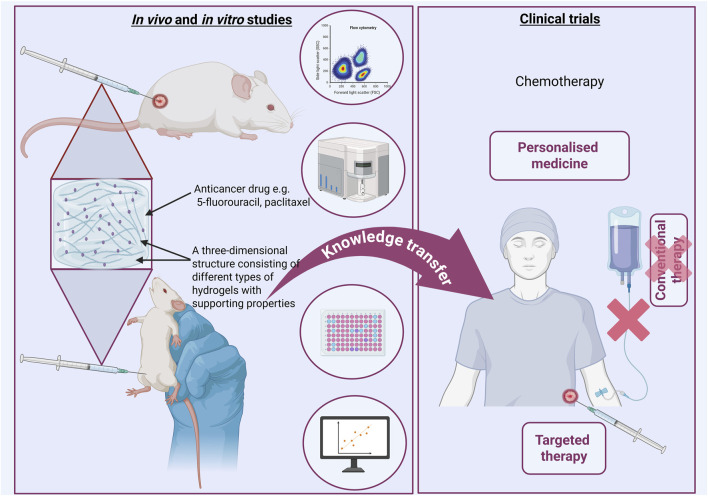
Intensive research in basic sciences is a real foundation for using their achievements in clinical trials. Perhaps in the near future it will be possible to use these achievements in practice. Created with BioRender.com.

The development of hydrogels in oncology in the coming years will dynamically evolve towards personalized therapy and intelligent therapeutic systems (Lee et al., 2025; Liu et al., 2025) (Figure 6). Their further development towards modification may change the perspective on cancer treatment methods. It is important to support interdisciplinary research, especially combining basic science with practical knowledge transfer in clinical practice.
